# Acceptability of a Mobile Health Behavior Change Intervention for Cancer Survivors With Obesity or Overweight: Nested Mixed Methods Study Within a Randomized Controlled Trial

**DOI:** 10.2196/18288

**Published:** 2021-02-16

**Authors:** Jenny M Groarke, Janice Richmond, Jenny Mc Sharry, AnnMarie Groarke, Owen M Harney, Mary Grace Kelly, Jane C Walsh

**Affiliations:** 1 Centre for Improving Health-Related Quality of Life School of Psychology Queen's University Belfast Belfast United Kingdom; 2 Letterkenny University Hospital Donegal Ireland; 3 School of Psychology National University of Ireland, Galway Galway Ireland

**Keywords:** mHealth, self-management, text messaging, activity tracker, exercise, diet, overweight, obesity, cancer survivors, qualitative research, mobile phone

## Abstract

**Background:**

A significant proportion of cancer survivors have overweight or obesity. Although this has negative implications for health, weight management is not a standard component of oncology aftercare. Mobile health (mHealth) technology, in combination with behavior change techniques (BCTs), has the potential to support positive lifestyle changes. Few studies have been carried out with cancer survivors; therefore, the acceptability of these tools and techniques requires further investigation.

**Objective:**

The aim of this study is to examine the acceptability of a behavior change intervention using mHealth for cancer survivors with a BMI of 25 or more and to gather constructive feedback from participants.

**Methods:**

The intervention consisted of educational sessions and an 8-week physical activity goal setting intervention delivered using mobile technology (ie, Fitbit activity monitor plus SMS contact). In the context of a two-arm randomized controlled trial, semistructured interviews were conducted to assess the retrospective acceptability of the intervention from the perspective of the recipients. The theoretical framework for the acceptability of health care interventions was used to inform a topic guide. The interviews were transcribed and analyzed using thematic analysis. A quantitative survey was also conducted to determine the acceptability of the intervention. A total of 13 participants were interviewed, and 36 participants completed the quantitative survey.

**Results:**

The results strongly support the acceptability of the intervention. The majority of the survey respondents held a positive attitude toward the intervention (35/36, 97%). In qualitative reports, many of the intervention components were enjoyed and the mHealth components (ie, Fitbit and goal setting through text message contact) were rated especially positively. Responses were mixed as to whether the burden of participating in the intervention was high (6/36, 17%) or low (5/36, 14%). Participants perceived the intervention as having high efficacy in improving health and well-being (34/36, 94%). Most respondents said that they understood how the intervention works (35/36, 97%), and qualitative data show that participants’ understanding of the aim of the intervention was broader than weight management and focused more on moving on psychologically from cancer.

**Conclusions:**

On the basis of the coherence of responses with theorized aspects of intervention acceptability, we are confident that this intervention using mHealth and BCTs is acceptable to cancer survivors with obesity or overweight. Participants made several recommendations concerning the additional provision of social support. Future studies are needed to assess the feasibility of delivery in clinical practice and the acceptability of the intervention to those delivering the intervention.

**International Registered Report Identifier (IRRID):**

RR2-10.2196/13214

## Introduction

### Background

Over the last 20 years, cancer has increased worldwide [1]. Reassuringly, the survival rates are also increasing [2,3]. However, a significant proportion of cancer survivors have overweight or obesity [4]. Oncologic treatment side effects, such as loss of muscular strength, fatigue, and physical inactivity, contribute to weight management issues during cancer and survivorship [5]. Overweight and obesity are associated with all-cause mortality, increased risk for cancer development, and increased risk of development of secondary cancer or subsequent primary cancer in cancer survivors [6-8]. Healthy lifestyle behaviors, such as regular exercise and healthy diet, have the potential to reduce treatment-associated morbidity and mortality in cancer survivors [9]. Therefore, interventions that support cancer survivors’ management of lifestyle behaviors are imperative.

Mobile health (mHealth) involves the use of mobile technologies, such as smartphones, tablets, apps, and wearable activity trackers, to improve health care, health outcomes, and public health. mHealth has been associated with significant reductions in weight and BMI [10] and positive health behavior changes [11,12]. Therefore, mHealth tools have the potential to meet the need for interventions that support weight and lifestyle management and, at the same time, are low resource and cost effective. At the same time, it is critical that the design of mHealth interventions is based on theory and evidence [13]. Evidence from systematic reviews suggests that using behavior change techniques (BCTs) significantly increases the success of weight management programs [14]. Interventions, including a greater number of BCTs, were associated with greater changes in health behaviors [15]. This was also the case for digital interventions [16]. Although there are a good number of lifestyle interventions for cancer survivors using BCTs [14], there are fewer mHealth interventions that have adopted this evidence-based approach to development, in the sense that very few have included relevant BCTs [17,18].

Interventions that are evidence based and effective may not be deemed acceptable to the prospective recipients. Acceptability is a key construct in intervention evaluation and is explicitly recommended in the UK Medical Research Council and National Institute of Health Research guidance on evaluating complex interventions [19]. Acceptability is often inferred from behavioral measures (eg, uptake, adherence, and retention), with fewer studies using explicit measures of acceptability or gathering feedback through postintervention interviews or focus groups [20]. Sekhon et al [21] proposed a theoretical framework to advance the systematic assessment of the acceptability of health care interventions. They defined acceptability as a “multi-faceted construct that reflects the extent to which people delivering or receiving a healthcare intervention consider it to be appropriate, based on anticipated or experienced cognitive and emotional responses to the intervention” [21]. The framework takes into account constructs, such as the participant’s attitude toward the intervention, the burden of participating, and the participant’s understanding of the intervention and how it is intended to work (a full description is given in Textbox 1). This method of assessment comprising multiple constructs offers a more fine-grained approach to detecting the source of acceptability issues that can be used to improve future interventions [20].

**Definitions of the constructs in the theoretical framework of acceptability of health care interventions.**

Constructs of acceptability:

- Affective attitude: How an individual feels about the intervention
- Burden: The perceived amount of effort that is required to participate in the intervention
- Ethicality: The extent to which the intervention has good fit with an individual’s value system
- Intervention coherence: The extent to which the participant understands the intervention and how it works
- Opportunity costs: The extent to which benefits, profits, or values must be given up to engage in the intervention
- Perceived effectiveness: The extent to which the intervention is perceived to be likely to achieve its purpose
- Self‐efficacy: The participant’s confidence that they can perform the behaviors required to participate in the intervention

Qualitative methods add significant value to trial research [22,23], contributing feedback that would not otherwise be captured. For successful uptake and implementation, it is important that the content, design, and delivery of interventions are considered acceptable to the target group [24]. In the area of mHealth, many studies have focused on the usability of technologies rather than the acceptability of interventions that use mobile technology. Some studies have supported the acceptability of mHealth solutions for people living with and beyond cancer [25-28]. However, acceptability is often operationalized behaviorally as adherence and engagement with technology. Few studies in this area have moved beyond behavioral measures or adopted a broader qualitative approach to understanding acceptability [29,30]. Therefore, we currently lack a robust understanding of the acceptability of mHealth behavior change interventions for cancer survivors with obesity or overweight. This study is the first to apply the theoretical framework of acceptability [21] to systematically assess the acceptability of an mHealth intervention for this high-risk group.

### Aim

This study aims to examine the acceptability of the Moving On intervention to its recipients and to obtain constructive feedback on the intervention and participants’ recommendations for improving the intervention content or delivery. The actual trial of the intervention was completed before this study. Full details of the trial protocol are reported elsewhere [31], and this paper presents the results of the mixed methods analysis of the retrospective acceptability of the intervention.

## Methods

### The Moving on Study Protocol

The Moving On study is a 2-arm parallel randomized controlled trial (RCT) of an intervention for cancer survivors with a BMI of 25 or more (International Standard Randomized Controlled Trial Number (ISRCTN): 18676721). Participants were assessed for clinical outcomes (ie, anthropometric measurements and functional exercise capacity), psychological outcomes (eg, quality of life), and behavioral outcomes (ie, dietary behavior and physical activity) at baseline (T0), at postintervention (12 weeks later: T1), and at the 24-week follow-up (T2).

The intervention uses mHealth in combination with BCTs to increase physical activity and improve diet, thereby improving health and well-being outcomes. This is a complex intervention targeting individuals’ self-management of lifestyle behavior, which is described in detail elsewhere [31]. In short, the intervention had 2 components.

1. A 4-hour lifestyle education and information session (week 1) was delivered by health care professionals (3 physiotherapists, 1 dietician, and 1 clinical psychologist). The physiotherapists demonstrated a series of daily strengthening exercises and recommended schedules for moderate-intensity physical activity. The dietician advised participants to; reduce their calorific intake, reduce red meat, processed meat, salt, and sugar intake; and increase fruit, vegetable, and fiber intake. The clinical psychologist offered practical strategies for problem solving, identifying barriers to change, and preventing relapse. The BCTs included in this session were *goal setting (outcome), provide information on consequences of behavior to the individual, demonstration of the behavior, problem solving, goal setting (behavior),* and *action planning.*
2. An 8-week physical activity goal setting intervention (weeks 4-12) was delivered using mobile technology (ie, Fitbit activity monitor plus SMS contact). Participants received weekly messages with feedback on their average daily step count and a goal to increase their step count by 10% in the following week. The BCTs included in the personalized goal setting intervention were *self-monitoring* of behavior, *feedback* on behavior, *goal setting (behavior), graded tasks, social reward,* and *review behavior goal(s).*

The design of this intervention is well matched with the National Institute for Health and Care Excellence guidelines for weight management of people with obesity [32]. They identify multicomponent interventions as the treatment of choice, which should include effective behavior change strategies aimed at increasing physical activity or decreasing inactivity, improving dietary behavior and quality of diet, and reducing energy intake.

### Study Design

This study was a mixed methods study nested within an RCT. In the context of the RCT, postintervention semistructured interviews were conducted, and a brief quantitative survey was administered to assess the retrospective acceptability of the intervention from the perspective of the recipients. Response rates and retention rates for the full trial were also used as indicators of the acceptability of the intervention. The design of this study was approved by the Research Ethics Committee of the National University of Ireland, Galway, and by the Research Ethics Committee at Letterkenny University Hospital.

### Study Setting

The Moving On study was a nonblinded RCT implemented in the oncology outpatient department at Letterkenny University Hospital in County Donegal, Ireland. Participants were cancer survivors with a BMI of 25 or more who had completed active cancer treatment. For this study examining the acceptability of the Moving On intervention, only participants randomized to receive the intervention were eligible for participation.

### Recruitment

At the 24-week follow-up, all participants in the intervention condition were asked to complete a 5-item survey to provide feedback on the intervention. For the qualitative component, all participants in the intervention condition were included and invited to participate in the qualitative study. No exclusion criteria were applied. An invitation to participate was included with their appointment letter for the final 24-week assessment of the intervention. One week before the final assessment, the invitation letter was followed up with an SMS to the group. The participants were asked to contact the research team if they were interested in participating in the qualitative study. Interviews were conducted with all volunteers who were available to be interviewed at their 24-week assessment (ie, 12 weeks postintervention). Written consent was obtained from 13 participants. The sample size was primarily determined by the size of the participant pool (ie, the intervention group) and the availability of participants. Within the context of this study, the data collected were judged by the research team to be adequate in both amount and variety to answer the research question. The participants did not receive any compensation.

### Qualitative Methodology

Thematic analysis was conducted using the essentialist or realist approach (ie, reporting experiences, meaning, and the reality of participants). This is a semantic-level analysis that looks for explicit meanings in data [33]. All interviews were individual and face-to-face and were conducted in the study setting. The semistructured interview topic guide was based on the theoretical framework of acceptability of health care interventions (Textbox 1) [21]. The following constructs were deemed the most relevant and used to inform the topic guide: affective attitude, burden, intervention coherence, perceived effectiveness, and self-efficacy. Participants were also asked about their motives for participating in the study and to provide feedback and recommendations for improving the Moving On intervention. The topic guide is included in Multimedia Appendix 1.

### Quantitative Methodology

A 5-item self-report measure of acceptability was created for this study, corresponding to the 5 constructs of the theoretical framework of acceptability of health care interventions used to inform the qualitative topic guide. Using a 5-point Likert scale, participants in the intervention were asked to rate their satisfaction with the intervention (affective attitude), the amount of effort required to participate (burden), the extent to which they understand the intervention and how it is intended to work (intervention coherence), the perceived effectiveness of the intervention, and their confidence in performing the behaviors required to participate (self-efficacy). Response rates and retention rates for the trial were used as behavioral indicators of the acceptability of the intervention.

### Data Analysis

Descriptive statistics were used to analyze the quantitative data using IBM (International Business Machines corporation) SPSS Statistics 24. Interviews were audio recorded and transcribed professionally. Transcripts were imported into NVivo 12 for analysis. Following the procedure outlined by Braun and Clarke [33], the transcripts were initially read by the primary coder (JG) several times to become familiar with the data. JG and JM independently coded 2 transcripts and agreed upon initial codes. The remaining transcripts were coded by JG. The analysis followed a deductive approach guided by the theoretical framework of acceptability. Transcripts were read in full and coded line by line to identify units of meaning relevant or interesting to answering the research question. These initial codes were then grouped together into the 5 domains described in the theoretical framework of acceptability and into a theme reflecting recommendations to improve the intervention by a multidisciplinary team (JG, JM, JR, MK, JW, and AG). Finally, members of the research team with practice-based experience (JR and MK) reviewed all the transcripts in relation to the themes identified. After analyzing all the transcripts, the analytic team was satisfied that no further interviews were necessary as the codes and themes identified were sufficient to develop a thorough understanding of the acceptability of the intervention and, therefore, meet the aims of the study [34]. The rigor of this study was enhanced by the secondary coding carried out by a multidisciplinary team of 6 such that each transcript was reviewed by at least three team members.

## Results

### Participants

A total of 13 participants (2 males) were interviewed, and 36 participants completed the quantitative survey. Participant characteristics are described in Tables 1 and 2.

**Table 1:** Characteristics of the 13 participants interviewed in this study.

Participant ID	Gender	Age (years)	Years since diagnosis	Weight changes at 24-week follow-up (kg)
001	Female	50	>5	−12.4
002	Female	51	>5	−10.6
003	Female	66	2-5	−7.4
004	Female	53	2-5	−7.2
005	Female	53	2-5	−6
006	Female	66	>5	−4.8
007	Male	54	2-5	−3.2
008	Female	67	>5	−0.80
009	Female	58	>5	−0.20
010	Female	68	2-5	−0.00
011	Female	38	<2	+1.5
012	Male	60	2-5	+3.0
013	Female	43	>5	+11.6

**Table 2:** Descriptive characteristics of participants in this mixed-methods study and in the intervention group of the full trial.

Participant characteristics	Qualitative interview (n=13)	Quantitative survey (n=36)	RCT^a^ (n=53)
Age (years), mean (SD); range	55.92 (9.03); 38-68	56.55 (6.40); 41-68	55.61 (8.05); 30-68
Male, n (%)	2 (15)	7 (19)	12 (28)
Years since diagnosis, mean (SD); range	5.38 (3.42); 1-12	5.78 (3.52); 1-15	6.59 (3.13); 1-15
Weight change in kilogram at 24-week follow-up, mean (SD); range	−2.81 (6.36); −12.4 to +11.60	−2.15 (3.57); −12.4 to +11.60	−1.74 (4.48); −12.4 to +11.60

^a^RCT: randomized controlled trial.

### Qualitative Findings on Acceptability

In opening the semistructured interviews, participants were asked about their motives for participating in the trial. Participants were clear that interventions to improve the health and well-being of cancer survivors were both acceptable and necessary. One participant had felt “very traumatised” by the experience (010). Other participants reported living with and beyond cancer as “very difficult” (012) and felt isolated because people who have not had cancer “don’t...really understand” (007) what they have been through and what they continue to deal with. They emphasized the desire for continuing support during survivorship. They also described ongoing health and psychological issues and having intrusive thoughts and concerns about these issues:

After cancer it can be a very lonely place. After you go through your treatment and you go through because you are getting great help and great encouragement during the process but when you come out the other end it can be very lonely, daunting kind of a place because every ache and pain you get you might just go in the one direction...you do need that support.

003

Findings on the perceived acceptability of the Moving On intervention, developed through deductive analysis guided by the theoretical framework of acceptability, are presented under Theme 1: Acceptability, with 5 subthemes relating to the 5 aspects of the framework applied in this analysis. Theme 2 describes participants’ recommendations for improving the Moving On intervention.

#### Theme 1: Acceptability

##### Affective Attitude: How an Individual Feels About the Intervention

Participants held a positive attitude toward the intervention. They described feeling “delighted” (010) and feeling “glad” (007) and “grateful” (003) that they had taken part in the intervention. However, one participant described feeling “absolutely gutted” and “like I’d failed” when they had not lost weight. As a result, she reported feeling “quite isolated” (009):

I truly appreciate it...I don’t know where I would be only for it.

003

In relation to the mHealth components, 2 participants said they loved their Fitbit and others described it as “absolutely great” (003) and “very beneficial” (013):

The Fitbit was fantastic... It didn’t control me, but it was a tool that gave me the information I needed to adjust.

002

The text messages were a source of comfort, “that someone is there for you*...*caring for you” (006). On the other hand, 3 of the interviewees mentioned some negative or mixed feelings toward weekly text messages. The participant quoted above (002), who had felt empowered by Fitbit, felt that being prescribed a daily step count goal took away her self-control. Two other participants interpreted the encouragements to be more physically active as meaning they were not trying hard enough already and described feeling “angry” and “annoyed” (009) as a result:

At one stage I remember being angry, it was saying “you could do better.”

010

Attendance at the half-day lifestyle information and education session was described as “really good” (003) “very useful, very informative” (012). One participant saw it as a good opportunity to talk with other people who have been through the same experience of having cancer:

I think that is a good starter to launch you on your effort to try to improve. Yea, I would say that was the best part for me.

012

##### Burden: The Perceived Amount of Effort That is Required to Participate in the Intervention

Only 3 participants described participation as a lot of effort, and the remaining participants described it as limited effort and manageable, especially given their motivation to take part. One woman felt that for her survival she needed a “big, big healthy change,” and therefore, “big effort was required” (010):

No I don’t think it took a lot of effort. I felt very committed to it and I felt I wanted to do it.

003

Some wondered whether the burden of participating might be higher in the earlier stages of cancer survivorship. The majority of respondents said that changing their dietary behavior was the most difficult aspect of the intervention. Although the majority of participants saw improving diet as the most difficult aspect, increasing physical activity was deemed more time consuming. Participants reflected on external factors that made behavioral changes more challenging, specifically illness among family members and poor weather conditions. They also highlighted times when health behaviors were more difficult to maintain (ie, the weekend and during summer).

##### Intervention Coherence: The Extent to Which the Participant Understands the Intervention and How it Works

Weight reduction was an important outcome of the trial; however, only 5 participants referred to weight in their responses. For many participants, the primary aim of the intervention was to provide motivation. For others, the intervention was synonymous with motivation:

If I hadn’t got the motivation, the programme, I don’t know where I would be now you know...in terms of quality of life.

010

Aims such as improving mental health and well-being, fitness, eating better, living healthier, and increasing energy have been described. They also identified much broader aims, such as “moving forward” (006) and “getting your life back” (004). In relation to cancer survivorship, participants spoke about goals such as getting “back on track after treatment” (011), “easing back into life” (002), and preventing recurrence:

The goal of the programme in my head was to encourage people to be fitter and eat better to avoid illness [recurring] ultimately.

005

Participants recognized education as a key function of the intervention:

I think it’s really to make you aware, it builds your awareness and educates... I’ve learned a lot.

009

The mHealth intervention was perceived to work through a combination of self-monitoring and monitoring by the research team. Seeing progress, having a goal, having it recorded, receiving positive feedback, and being watched were highlighted by participants as mechanisms of health behavior change:

I suppose just having a target you know. And knowing I guess that if you did push it, it was being recorded and was coming back to you to say, “well done,” you know a bit of enthusiasm.

005

A number of participants considered the baseline and follow-up assessments as part of the intervention and discussed them in reference to how the intervention works, particularly in relation to their level of motivation leading up to the assessments:

I do think the little interviews and check-ups here really do keep you focused that little bit.

013

##### Perceived Effectiveness: The Extent to Which the Intervention is Perceived as Likely to Achieve its Purpose

Although some of the participants interviewed lost a significant amount of weight over the 6-month study (3.2-12.4 kg), weight loss did not feature strongly in their descriptions of the effects of the intervention. The majority of participants mentioned feeling physically fitter, having more energy, sleeping better, and feeling less fatigued:

My goal was to lose weight and get fitter and now I have achieved that.

001

Almost all participants mentioned some emotional and psychological improvement, such as, feeling “good – physically, mentally, spiritually...so happy” (003), “mentally a lot better...calmer” (013), and more “productive” (005):

Certainly the mood swings are not as hectic...I have come out the other end feeling way more positive than I would have been. I would have been an emotional wreck last year.

013

There were also changes related to self and self-image. One woman who lost a lot of weight now felt more confident and noticed a change in her behavior:

Before I would have just stayed in the background...now I put myself forward to do things.

001

For one woman, the intervention was the first step in reclaiming her old self and how she used to look before cancer:

With being sick, and with everything that happened, I didn’t feel me, so this sort of put me on the first step to start losing weight, and getting more to looking like myself, by the time I lost a bit of weight, I had some hair got, and you know, standing looking in the mirror thinking “this is what you used to look like...6 months ago I didn’t look like this”.

004

##### Self-Efficacy: The Participant’s Confidence That They Can Perform the Behaviors Required to Participate in the Intervention

Responses were evenly mixed in relation to how confident participants were that they would be able to perform the behavior required to participate in the intervention (ie, improve their diet and increase physical activity). Participants described these changes as something that they wanted to do but needed motivation. Others were not sure of their capability at the outset of the intervention; however, their confidence grew over time:

What seemed like a problem at the beginning of the journey you have so many solutions to it by the end of the journey.

003

The text messages and goals were the support most commonly referred to as helpful for promoting self-efficacy. Participants also discussed the different sources of social support they received throughout the intervention from staff, friends, family, and other participants:

Well I got support from home too. I got support from here [the hospital]... People at work supported you too because they started to join ya. They were starting getting Fitbit and walking and things...I got a lot of support from everybody.

001

Participants spoke about having trust in the health information and behavior change advice because of its source, which was the local hospital cancer team and the behavioral science research team. This provided them with confidence in changing their behavior:

The fact that it is linked into the hospital is very helpful and very reassuring.

003

#### Quantitative Findings on Acceptability

##### Recruitment and Retention Rates

The high response rate for the full trial (123/159, 77.4%) and high retention rate (53/62, 85%) of participants in the intervention arm support the acceptability of the intervention. Of those who declined to participate in the trial, 77% (28/36) were not interested, 14% (5/36) declined for health reasons, and 8% (3/36) declined because of practical constraints (eg, childcare responsibilities). Of those who dropped out of the intervention arm of the trial (9/62, 15%), 7 participants were lost after the baseline assessment because of rescheduling of the lifestyle information and education session at short notice because of adverse weather conditions. One participant passed away (unrelated to the trial) between the 12- and 24-week assessments, and one participant did not attend the 24-week follow-up assessment.

##### Acceptability Survey

All 53 participants in the intervention condition who attended their 24-week follow-up assessment were asked to complete an additional 5-item survey to provide feedback on the intervention, and 68% (36/53) of participants completed the survey. Results show that 97% (35/36) of the participants surveyed were satisfied or very satisfied, indicating a positive affective attitude toward the intervention. The same proportion of participants reported that the intervention had a high coherence (35/36, 97%). Furthermore, 94% (34/36) of respondents believed that the intervention was effective in improving health and well-being outcomes. There was more variability in responses to questions of burden, with a similar proportion of participants reporting that the intervention required a great deal of effort (6/36, 17%) as those reporting it required very little effort (5/36, 14%). There was also some variability regarding participants’ self-efficacy; however, 83% (30/36) of the participants reported being confident that they performed the behaviors necessary for the intervention. All results are presented in Multimedia Appendix 2.

##### Theme 2: Recommendations for Improving the Moving on Intervention

When asked how the intervention could be improved, a number of participants wanted more frequent contacts and easier access to clinicians. Although participants wanted more contact, many spoke of the availability of support from staff if required. This was especially valuable posttreatment when people reported feeling isolated and concerned about their health. One participant felt it would be useful to have another session at the end of the intervention to get remotivated:

I made all these changes and they’re not working...I’d like to have spoken to somebody about that.

009

Some participants wanted less information presented during the lifestyle information and education session, whereas others wanted more information. For instance, one participant wanted more education on diet and portion size. Another recommended dietary reminders or ongoing goal setting in relation to healthy eating. One participant recommended providing more training on how to use Fitbit. In relation to the goal setting SMS, 2 participants recommended having more SMS over a longer duration, perhaps in declining frequency. Another participant recommended that SMS text messages be delivered on a fixed schedule (ie, on the same time and day each week).

Participants recommended a range of strategies to increase opportunities for interaction and social support among the participants, for example, a longer time frame for the lifestyle information and education session for people to get to know one another and a group debrief at the end. One participant recommended including peer support that could take the form of an online support group and that participants may actually get involved in delivering elements of the intervention (ie, goal setting). Another participant recommended assigning people a walking partner so they could motivate each other and a half-day session at the midway point where participants could discuss their progress, or lack thereof, with the aim of reigniting their motivation.

## Discussion

### Principal Findings

The results are consistent with constructs from the theoretical framework of acceptability of health care interventions [21], suggesting high acceptability of the intervention. Overall, the mixed methods results indicate that the majority of participants were satisfied and had a positive attitude toward the intervention, with high perceived effectiveness and high coherence. Most participants were confident that they could perform the required behaviors for the intervention, and qualitative data showed that the design of the intervention helped build self-efficacy in performing health behaviors. In both quantitative and qualitative responses, there was greater individual variability in participants’ determinations of the intervention’s level of burden and participants’ sense of self-efficacy. The high response rate and low rate of attrition in the trial support these qualitative and quantitative findings. Therefore, we are confident that this intervention using mHealth and BCTs to improve health and well-being outcomes is acceptable to recipients. The findings of this study are consistent with research using quantitative and qualitative methods that have found that mobile and digital health solutions are acceptable to people living with and beyond cancer [25,27,28,30] and studies supporting the acceptability of health behavior change interventions more broadly [35,36].

Participants reported a high level of satisfaction with the intervention. Most participants held a positive attitude toward the intervention and enjoyed many of its elements. In particular, mHealth components were rated positively. In terms of intervention coherence, participants’ understanding of the aim of the intervention was less focused on weight management and more focused on *moving on* psychologically from cancer. The intervention was perceived to have high efficacy, and the participants described the effects in different areas, especially physical health and fitness, mental health and well-being, and self and self-image. Although weight loss was an important outcome of the intervention, it is noteworthy that weight loss did not feature strongly in participants’ responses in terms of the effects of the intervention. This may be linked to the finding that many participants judged the goal of the intervention to be much broader than weight loss. Discordance between patient and provider priorities with regard to health goals and treatment outcomes has been previously observed in the management of cancer and other chronic conditions [37-39]. This highlights the importance of patient-centered care [40], along with the person-centered design of health care interventions [41]. There was a variety of responses in terms of the level of burden posed by participating in the intervention and participants’ confidence that they could perform expected behaviors. The low level of attrition implies that participating in the intervention itself was not particularly onerous or burdensome; however, the qualitative responses reveal how challenging health behavior change is on a personal level. This is evident in the low success rate of behavioral weight loss interventions [42]. mHealth tools may support health behavior change [43,44]; however, these tools alone do not necessarily increase self-efficacy or reduce the burden of changing well-established dietary and physical activity behaviors.

Participants were generally very positive about the content and delivery of the intervention. Most of the recommendations for improving the Moving On intervention focused on increasing the frequency, duration, and scope of the existing intervention components. For example, the goal setting intervention focused on increasing physical activity, and participants recommended that we extend this goal setting to dietary behavior. This is not unexpected, given reports that dietary behavior change was deemed the most difficult aspect of the intervention, and perhaps, this was because of them receiving less behavioral support in this respect. Clinical guidelines for obesity management recommend that interventions target both physical activity and diet [32,45]. Interventions using goal setting have typically focused on either diet or physical activity, rather than targeting both simultaneously [46]. However, goal setting has been used in interventions focusing on dietary behavior change with positive effects [47]. Participants recommended longer and additional lifestyle information and education sessions, with more time to interact with health care professionals and more goal setting SMS over a longer duration in declining frequency. There are a limited number of mHealth interventions using BCTs with cancer survivors; however, one previous study found that behavioral prompts sent by email in declining frequency over 12 weeks significantly increased physical activity in survivors of breast cancer [18].

It is clear from the feedback that participants also wanted more opportunities for social interaction and support from other participants during the intervention. This is consistent with the findings of another study on breast cancer survivors and their preferences for wearable activity trackers. Their recommendations were to incorporate online and in-person peer support and doctor monitoring [29]. Social support is a common component of psychosocial interventions aimed at improving emotional adjustment in people with cancer [48,49]. The Moving On intervention aimed to improve health and well-being outcomes by improving health behavior. However, the findings presented here highlight the broader issues that cancer survivors face. Participants’ recommendations for increased social support are in keeping with participants’ descriptions of the cancer experience as lonely and isolating. Together, these findings suggest that this mHealth behavior change intervention, although acceptable for self-management of lifestyle behavior, may not be sufficient to meet the full range of physical and psychological health needs of people living beyond cancer. Future research will be needed to determine whether the addition of social support components to the Moving On intervention has an incremental benefit on the effectiveness of the intervention.

Qualitative research can inform the development of future trials and interventions. Participants’ responses raised important questions about the ideal timing of the Moving On intervention. Some felt the burden of participating would be higher in the earlier stages of cancer survivorship when fatigue and treatment effects were most pronounced [50]. At the same time, others described feeling lost and afraid when their acute cancer treatment was complete and their follow-up would be an outpatient visit every 6-12 months, supporting the idea that this may be a good time to intervene. Participants also identified times when behavior change was more difficult, opening up the potential for adapting the mHealth intervention as a just-in-time intervention [51]. These are complex, technologically advanced interventions that provide real-time behavioral support, for example, when the participant has an opportunity to engage in a health behavior or is likely to experience a trigger for unhealthy behavior. A recent review found that these interventions are acceptable to participants and are often used in combination with the same BCTs as in this study (ie, goal setting, prompts, feedback, and action planning) [52].

Qualitative findings can also inform the interpretation of the results of RCTs. The assessment of study outcomes at baseline, at 12 weeks, and at 24 weeks were not coded as BCTs; however, based on the results of this study, they may have been important motivators of behavior change. It is possible that future iterations of the intervention that do not include assessment of clinical, psychological, and behavioral outcomes would be less effective. Our intention was that the goal setting intervention would motivate participants to increase their activity level. However, these qualitative results suggest that participants’ motivation was influenced by multiple factors, including self-monitoring their activity via Fitbit, an awareness of being monitored by the research team, accumulating self-efficacy, encouraging interactions with staff (at the lifestyle information and education session and SMS contact), and attending assessments.

Although not an explicit focus of this study, a small number of issues relevant to fidelity were described by participants during the interviews. Some participants spoke of being enrolled in other programs (eg, mindfulness courses and sports clubs), and although this was not discouraged, it was not measured or controlled for. Another participant revealed that he had broken his Fitbit and started using a different service (MyFitnessPal) without informing the research team. Other participants used the Fitbit app in ways they had been explicitly asked not to (ie, for food logging and calorie counting). Fidelity is an important aspect of evaluating complex interventions [53] and may be relevant to the acceptability of an intervention, but it was not measured explicitly in the trial and would be useful to consider in future testing of similar interventions.

Acceptability is an aspect of feasibility. However, future studies are needed to assess the feasibility of delivering this intervention in clinical practice. Although the intervention was perceived as low burden by participants, the burden of delivering the intervention in routine care may be high for clinical staff. There is significant promise of and potential for mHealth to limit the burden on the health care system [13]. However, the findings of this study suggest that in addition to mHealth solutions, cancer survivors want additional and extended support from their health care providers in relation to self-management of lifestyle behavior. In many clinical contexts, this may be challenging; however, this program could be facilitated external to the hospital setting, and advancements in digital health technology make this more of a possibility than ever before.

### Limitations

This study used qualitative research to assess the retrospective acceptability of the Moving On intervention. The *person-based approach* recommends in-depth qualitative research at the planning and development stage to enhance the acceptability and feasibility of interventions [41]. Unfortunately, participants’ perspectives were not considered during the design phase of the intervention. Reassuringly, the results of this study suggest that the Moving On intervention was seen as acceptable by those who participated in it. Relative to other qualitative studies in health research [54], the sample size was limited. At the same time, a sizable portion of the intervention group was interviewed (13/53, 24%). Furthermore, there was a limited number of male participants; however, this is reflective of the composition of the sample in the full trial. With regard to acceptability, the qualitative responses were consistent with the quantitative data with a larger sample size of 36 (representing 36/53, 68% of the intervention group). This concordance provides further support for our judgment that the number of interviews was sufficient to meet the goals of the analysis. Finally, this study focused on the acceptability of the intervention to those receiving the intervention only. It would be equally interesting and important to assess the acceptability of the intervention to those responsible for delivering it.

### Conclusions

This study provides insight into the experiences of participants enrolled in the Moving On intervention. This mixed methods analysis identified that the intervention was acceptable to the participants. Participants spoke of cancer survivorship as lonely and isolating, and they wanted more social support and peer interaction to be incorporated into the intervention. Overall, participants were very positive about the intervention in terms of both content and delivery. As it was acceptable and was perceived as having the broad goal of *moving on* psychologically, if found to be effective, the intervention could be offered to individuals following completion of their active cancer treatment as part of standard care to promote the health and well-being of survivors.

## Appendix

Multimedia Appendix 1 Semistructured interview topic guide.

Multimedia Appendix 2 Results of quantitative survey of acceptability.

## Abbreviations

BCT: behavior change technique
mHealth: mobile health
RCT: randomized controlled trial
