# Supplementary material for: Acceptability of a Mobile Health Behavior Change Intervention for Cancer Survivors With Obesity or Overweight: Nested Mixed Methods Study Within a Randomized Controlled Trial
Source: JMIR Mhealth Uhealth. 2021 Feb 16;9(2):e18288. doi: 10.2196/18288 (PMC7925146; doi:10.2196/18288)
Supplement: Multimedia Appendix 1 [file mhealth_v9i2e18288_app1.doc]

**Research study: Examining the Acceptability of the Moving On Intervention.**

**Briefing:**

**1) Thank participant for agreeing to take part.**

**2) Introduce self.**

**3) As described in the Participant Information Sheet we are interested in** hearing about your experience of participating in the Moving On intervention, as well as, collecting feedback on the programme and how we might improve it.

**4) If at any time during the interview you do not wish to answer a question that’s okay.**

**5) I would like to record our conversation. The recording will be typed out, but everything you say will be anonymous. Your name and any names or places you mention will be taken out, so that if someone read your interview they would not know who you are.**

**6) If, at any stage, you wish to stop the audio recorder, please let me know.**

**7) Do you have any questions?**

**Topics to be explored: Below is a list of questions to be discussed in this study. The work will remain flexible with respect to participants’ agendas but we will cover the main topics outlined below. It is common in semi-structured work to develop topics and questions as new ideas emerge from early data collection. Therefore, we may add new topics as the interviews progress and data collection continues. However, the key topic of acceptability [topics 2-6] will remain the focus of the interview.**

1. **Experience of cancer survivorship**
   - When did your treatment end?
   - How did cancer treatment impact your health and wellbeing now? (e.g., your weight)
   - Why did you volunteer to participate in this program?
2. **Affective Attitude**; How an individual feels about the intervention

- How do you feel about the intervention?
- Did you enjoy taking part in the intervention?
- What did you enjoy about it? (e.g., personalised advice from experts at the info session, self-monitoring via Fitbit, motivation from goal-setting SMS?

1. **Perceived Effectiveness;** the extent to which the intervention is perceived as likely to achieve its purpose

- Did you experience any improvements in your health over the last 6 months?
- Did you experience any improvement in your wellbeing over the last 6 months?
- Did the intervention have any effect on your health and wellbeing?

1. **Self-Efficacy;** The participant’s confidence that they can perform the behaviour(s) required to participant in the intervention

- What action was required of you to participate in the intervention? (e.g. attend edu session, self-direct healthy diet, use fitbit, read SMS, increase step count +10%)
- Before the intervention how confident were you that you could carry out these actions?
- Did any aspects of the intervention increase your confidence that you could carry out these actions?
- What kind of support did you need to carry out these actions?

1. **Burden;** The perceived amount of effort that is required to participate in the intervention

- How much effort did you have to make to take part in the intervention?
- What was most difficult about taking part in the intervention?
- What took up most of your time when participating in the intervention?

1. **Intervention Coherence;** the extent to which the participant understands the intervention and how it works

- How would you describe the intervention, in terms of how you think it works?
- What do you think is the goal of the intervention?
- How do you think the intervention works?

1. **Recommendations**
   - What was good about the support you received/what could be improved on?
   - What in their view is the ideal support/treatment for someone struggling with their weight?
   - Do they have any personal recommendations to help others?
2. **Summary**
   - Was there anything I left out?
   - Anything else you would like to tell me
