# Supplementary material for: Acceptability of a Mobile Health Behavior Change Intervention for Cancer Survivors With Obesity or Overweight: Nested Mixed Methods Study Within a Randomized Controlled Trial
Source: JMIR Mhealth Uhealth. 2021 Feb 16;9(2):e18288. doi: 10.2196/18288 (PMC7925146; doi:10.2196/18288)
Supplement: Multimedia Appendix 2 [file mhealth_v9i2e18288_app2.docx]

The proportion and number of responses to a quantitative measure of acceptability (N=36).

|  |  |  |  |  |  |
| --- | --- | --- | --- | --- | --- |
| Affective attitude: How satisfied are you with the intervention? | Very dissatisfied | Dissatisfied | Neither | Satisfied | Very satisfied |
|  | — | 3% (1/36) | — | 19% (7/36) | 78% (28/36) |
| Burden: How much effort was required of you to participate in the intervention? | Very little | Not much | Some | Much | A great deal |
|  | 14% (5/36) | 17% (6/36) | 39% (14/36) | 14% (5/36) | 17% (6/36) |
| Intervention coherence: I understand how the intervention is supposed to work. | Strongly disagree | Disagree | Neutral | Agree | Strongly agree |
|  | — | 3%  (1/36) | — | 42% (15/36) | 55% (20/36) |
| Perceived effectiveness: I believe the intervention is effective for improving health and well-being. | Strongly disagree | Disagree | Neutral | Agree | Strongly agree |
|  | 3%  (1/36) | — | 3% (1/36) | 28% (10/36) | 67% (24/36) |
| Self-efficacy: I am confident I performed the behaviors required by the intervention. | Strongly disagree | Disagree | Neutral | Agree | Strongly agree |
|  | — | 3%  (1/36) | 14% (5/36) | 53% (19/36) | 30% (11/36) |
